# Detection of 3D curved trajectories: the role of binocular disparity

**DOI:** 10.3389/fnbeh.2013.00012

**Published:** 2013-02-20

**Authors:** Russell S. Pierce, Zhang Bian, Myron L. Braunstein, George J. Andersen

**Affiliations:** ^1^Visual Cognition and Human Performance Laboratory, Department of Psychology, University of CaliforniaRiverside, CA, USA; ^2^Department of Cognitive Sciences, University of CaliforniaIrvine, CA, USA

**Keywords:** 3D motion perception, binocular disparity, trajectory, curved trajectory, 3D perception, motion in depth

## Abstract

We examined the ability of observers to detect the 3D curvature of motion paths when binocular disparity and motion information were present. On each trial, two displays were observed through shutter-glasses. In one display, a sphere moved along a linear path in the horizontal and depth dimensions. In the other display, the sphere moved from the same starting position to the same ending position as in the linear path, but moved along an arc in depth. Observers were asked to indicate whether the first or second display simulated a curved trajectory. Adaptive staircases were used to derive the observers' thresholds of curvature detection. In the first experiment, two independent variables were manipulated: viewing condition (binocular vs. monocular) and type of curvature (concave vs. convex). In the second experiment, three independent variables were manipulated: viewing condition, type of curvature, and whether the motion direction was approaching or receding. In both experiments, detection thresholds were lower for binocular viewing conditions as compared to monocular viewing conditions. In addition, concave trajectories were easier to detect than convex trajectories. In the second experiment, the direction of motion did not significantly affect curvature detection. These results indicate the detection of curved motion paths from monocular information was improved when binocular information was present. The results also indicate the importance of the type of curvature, suggesting that the rate of change of disparity may be important in detecting curved trajectories.

The perception of the 3D motion trajectory of objects is an important visual task. Accurately judging the 3D motion trajectory of objects is important for activities such as intercepting an object or catching a ball. Previous research has examined three types of information used to recover the 3D trajectory of moving objects: (1) changes in binocular information, (2) changes in the angular speed and angular size of an object's projected image, and (3) background scene information which can be used to determine depth and layout of objects in a scene. In the present study we examined the use of binocular information and changes in angular speed and angular size for determining the trajectory of moving objects on curved paths.

As an object travels in depth, at least two sources of binocular information are available to specify its motion trajectory (Regan, 1993; Cumming and Parker, 1994; Rokers et al., 2009). One source of information is the changing disparity over time (CDOT) of an object relative to a fixed object or background (e.g., Beverley and Regan, 1974). A second source of information concerns binocular velocity information. As an object moves in depth it projects different image velocities to the two eyes. The visual system thus could combine the motion signals from both eyes and determine the speed and direction of motion by determining how this interocular velocity difference (IOVD) changes over time. Therefore, another source of information for the specification of motion trajectories may be IOVD (e.g., Welchman et al., 2008; but also see Lages and Heron, 2008).

Previous research has examined both of these sources of binocular information for the perception of motion in depth (e.g., Regan, 1993; Cumming and Parker, 1994; Shioiri et al., 2000; see Harris et al., 2008 for an excellent review as well as neurophysiological evidence of the mechanisms involved in the perception of motion in depth). For example, Cumming and Parker (1994) showed observers stimuli with random dot stereograms that were either temporally uncorrelated or temporally correlated. In the temporally uncorrelated stimuli, the IOVD was not available and only the rate of change in binocular disparity could be used to determine motion in depth. In the temporally correlated stimuli, both sources of binocular information were available. They found that the threshold to detect motion in depth was lower for the temporally uncorrelated stimuli as compared to temporally correlated stimuli, suggesting that the rate of change in binocular disparity was used to determine motion in depth while the IOVD was not useful. Similar findings were obtained in other studies (e.g., Lages et al., 2003). However, Shioiri et al. (2000) used binocularly uncorrelated random dot kinematograms and found that observers were able to discriminate motion in depth at high contrast, providing support for the use of IOVD in judging motion in depth. These studies, taken together, indicate that binocular information is used for the perception of motion in depth and suggest that binocular information may be important for the perception of curved motion paths.

In addition to binocular information, the observer has available monocular information for determining the 3D trajectory of a moving object. This information includes the change of angular speed and angular size of an object's projection as it travels in depth. Previous research has examined the usefulness of monocular information for determining the trajectory of a moving object (e.g., Todd, 1981; Regan and Kaushal, 1994; Regan and Gray, 2000; Harris and Drga, 2005; Rushton and Duke, 2007). For example, Todd (1981) proposed a mathematical model demonstrating that various characteristics of a motion trajectory, such as time to passage and angle of approach, could be derived from visual information in optic flow. Regan and Kaushal (1994) proposed that the direction of motion in depth could be specified by the ratio of the projected translational velocity to the rate of expansion of an approaching object. The results of this research indicate that observers are able to discriminate the direction of motion trajectories even when the direction and velocity of retinal image translation were removed as reliable cues.

As discussed above an extensive literature exists regarding the perception of motion in depth including the perception linear motion trajectories in 3D space. However, in the real world objects do not move exclusively along linear paths. Objects can move along curved paths. In the present study, we examined the ability of observers to detect motion of an approaching object on a constant curved path. Specifically, we investigated the use of binocular and monocular information for the detection of a curved path. Binocular information included the rate of change in binocular disparity and IOVD. Monocular information included a change of angular speed and angular size as the object approaches the viewpoint of the observer. Recently Zhang et al. (2012) found that judgments of the sign and magnitude of the curvature for curved trajectories at eye level were based primarily on changes in angular size rather than in angular speed. The primary purpose of the current study was to examine the ability of observers to detect curved motion paths from monocular information sources, and assess whether binocular information improved detection performance. In Experiment 1 we examined the effect of viewing condition (binocular vs. monocular) and type of curvature (concave vs. convex) on the perception of motion trajectories.

## Experiment 1

### Methods

#### Observers

Twelve college-age observers at the University of California, five males and seven females, who scored 8 or higher on the RandDot Stereotest, were unfamiliar with the purpose of the experiment, and were paid for their participation.

#### Apparatus

Stimuli were presented on a 20″ (51 cm) diagonal, 4:3 aspect ratio ViewSonic P225F 120 Hz CRT monitor operating at a resolution of 1024 × 768 pixels driven by a Dell Precision computer equipped with an NVidia Quadro graphics card. Custom experimental software was written in C++ and OpenGL using a Quad Buffered rendering technique compatible with StereoGraphics CrystalEyes three shutter glasses. The observer sat at a distance of 960 mm from the monitor and viewed the display through a collimating lens to minimize flatness information from accommodation (Andersen et al., 1998).

#### Design

The independent variables were viewing condition (binocular vs. monocular) and type of curvature (concave vs. convex). Four combinations between the two independent variables were manipulated between experimental blocks according to a Latin square. That is, the four conditions were arranged in a 4 × 4 Latin square and each observer went through one row in the square. The extent of curvature was adjusted within blocks according to a BestPEST staircase algorithm to determine the 75% threshold of discrimination between linear and curved trajectories (Lieberman and Pentland, 1982). Observers received trials until the BestPEST algorithm indicated that a threshold had been found (*M* = 71.02 trials, *SD* = 29.15 trials).

#### Procedure

**Figure 1:**
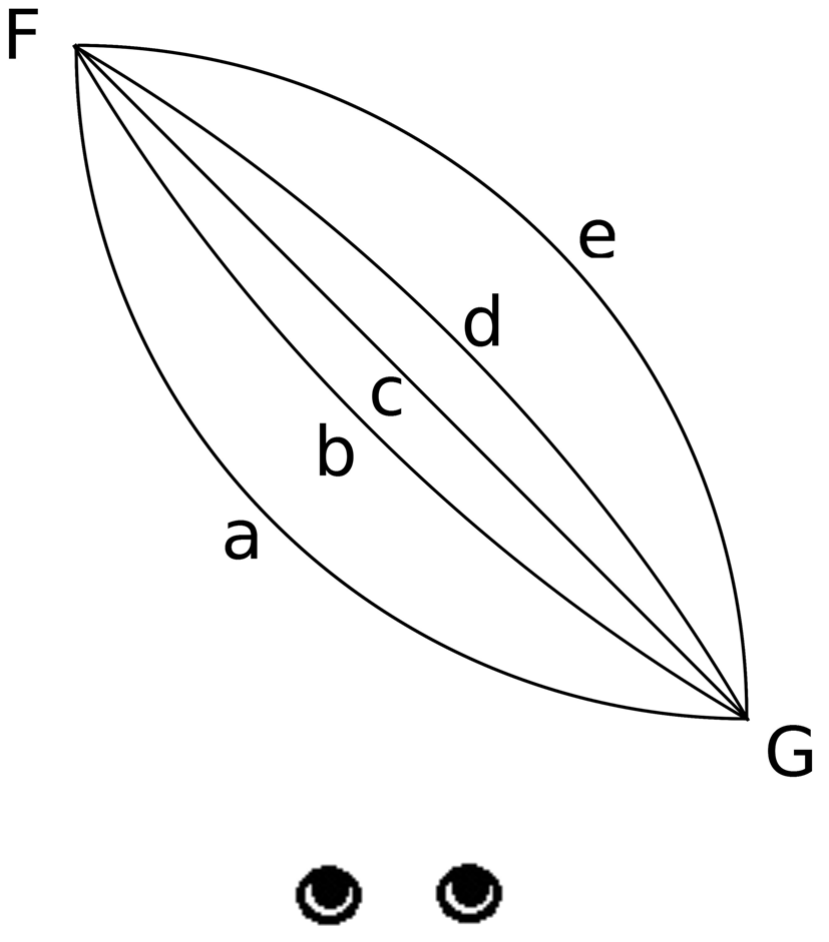
**A top view of two convex trajectories differing in magnitude of curvature (a and b), one linear trajectory (c) and two concave trajectories differing in magnitude of curvature (e and d) with the same magnitude as trajectories (a) and (b), respectively.** In Experiment 1, the object moved from position F to position G. In Experiment 2, the object either moved from position F to G or moved from position G to F. The orientation of the diagonal path was the same in both experiments.

The experiment was run in a dark room. The observers viewed the display through shutter glasses with their head position fixed by a chin rest. Observers in the monocular condition wore an eye patch over one of the lenses of the shutter glasses. On each trial, observers viewed two sequentially presented displays simulating motion trajectories of a purple sphere (illuminated from above in the simulation to produce shading on the sphere) traversing a medium gray background at eye level. In one display, the sphere moved along an oblique (45° angle) linear trajectory toward the observer (Figure 1, trajectory c). In the other display, the sphere traveled from the same starting and ending position along a curved trajectory along either a convex (Figure 1, trajectory a or b) or concave path (Figure 1, trajectory d or e). The simulated start point of the motion paths was 223 mm (9.15° visual angle) to the left of the observer at a simulated depth of 1385 mm (see Figure 1, point F). The simulated end point of the motion paths was 153 mm (8.63° visual angle) to the right of the observer at a simulated depth of 1008 mm (see Figure 1, point G). Therefore, the total simulated travel distance of the sphere was always equal in the horizontal and depth dimensions. The duration of each motion path was 3 s. When the sphere followed a linear path, it traveled between these two points at a constant 3D velocity. When the sphere followed a curved path, it traveled between these two points along an arc segment of a circle that intersected the start and end points of the linear motion path. The simulated horizontal and depth velocities varied along the motion path consistent with the curved path specified by a constant change in angular displacement relative to the center of curvature. The temporal order of the paths (linear or curved) was randomized on each trial. The maximal disparity change between any two stereo paired frames was 0.022°. Although there were no other objects on the display, the frame of the display was visible and could be used to establish relative disparity. Observers were given no instructions regarding eye movements and were free to move their eyes during the trial. Observers pressed the 1 key on the numeric keypad if the first motion path was curved and pressed the 2 key on the numeric keypad if the second motion path was curved.

### Results

**Figure 2:**
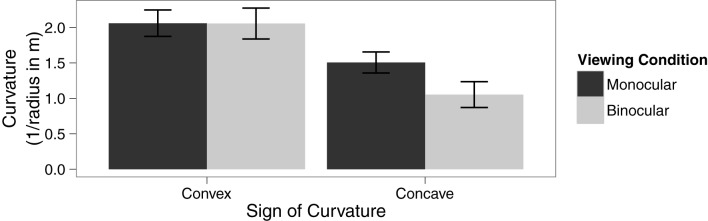
**Means in each condition with error bars representing ±1 between-subjects standard error of the mean**.

A within-subjects ANOVA was conducted to analyze the effect of viewing condition and type of curvature on the 75% threshold of discrimination between linear and curved trajectories. The main effect of viewing condition was not significant, *F*_(1, 11)_ = 4.69, *p* = 0.053, η^2^_G_ = 0.03. There was a significant main effect of type of curvature, *F*_(1, 11)_ = 12.22, *p* = 0.005, η^2^_G_ = 0.29. However, these effects were qualified by a significant interaction between these factors, *F*_(1, 11)_ = 5.52, *p* = 0.04, η^2^_G_ = 0.03, see Figure 2. Specifically, paired sample *t*-tests reveal that observers were able to make use of binocular information in detecting concave motion paths, *t*_(11)_ = 2.63, *p* = 0.02, but not for convex motion paths, *t*_(11)_ = 0.04, *p* = 0.97.

## Experiment 2

The results of Experiment 1 indicate that disparity information can be used for the detection of concave motion paths. However, the results did not indicate that disparity information was useful for convex motion paths. There are several possible reasons why disparity information was not useful for this condition. At the simulated distances studied in Experiment 1, the rate of disparity change in convex paths may not have been sufficient for discriminating between a convex path and a linear path. Also in Experiment 1, all objects were approaching in depth. This meant that for concave motion paths the greatest disparity change occurred toward the end of the motion path, whereas for convex motion paths the greatest disparity change occurred close to the start of the motion path. Given that the motion path started at zero velocity, it is possible that observers may have had difficulty using disparity information following the onset of motion.

To test both of these possibilities we reduced the distance between the observer and the motion path, thereby increasing the rate of disparity change for both convex and concave trajectories. If the inability to find an effect of disparity for convex paths in Experiment 1 was due to the lower rate of change in disparity, then repositioning the stimuli to a closer distance should increase the utility of disparity information for both convex and concave trajectories. We also varied the direction of motion (approaching vs. receding). If the reduced effect of disparity for convex motion paths was due to the sudden onset of motion occurring when disparity change was greatest then presenting receding motion will remove this factor, resulting in the greatest disparity change occurring near the end of the motion sequence.

### Method

#### Observers

Sixteen college-age observers at the University of California, Riverside, seven males and nine females, who scored 8 or higher on the RanDot test of stereo vision, were unfamiliar with the purpose of the experiment, and were paid for their participation.

#### Design

The independent variables were viewing condition (binocular vs. monocular), type of curved trajectory (concave vs. convex), and direction of motion (approaching vs. receding). All eight combinations between the independent variables were manipulated between experimental blocks according to a Latin square. That is, the eight conditions were arranged in an 8 × 8 Latin square and each observer went through one row in the square. The magnitude of curvature was adjusted within blocks according to a two-up one-down staircase procedure to reduce the variability in the number of trials required to reach a threshold. Specifically, the average of 10 reversal points was used to determine the threshold of discrimination between linear and curved trajectories (*M* = 85.43 trials, *SD* = 12.70 trials).

#### Procedure

As in Experiment 1, the observer sat at a distance of 960 mm from the monitor and viewed the display through a collimating lens to minimize flatness information from accommodation (Andersen et al., 1998). Approaching motion paths moved in the same manner as in Experiment 1. Receding motion paths started close to and to the right of observers (point G, Figure 1), and traveled further away from the observer and to their left (point F, Figure 1). In order to produce motion paths similar to those in Experiment 1 the simulated position of the point F was 226 mm (10.41° visual angle) to the left of the observer at a simulated depth of 1230 mm, and the simulated position of point G was 156 mm (10.42° visual angle) to the right of the observer at a simulated depth of 848 mm. The maximal disparity change between any two stereo paired frames was 0.031°. In all other respects the procedure was identical to Experiment 1.

### Results

**Figure 3:**
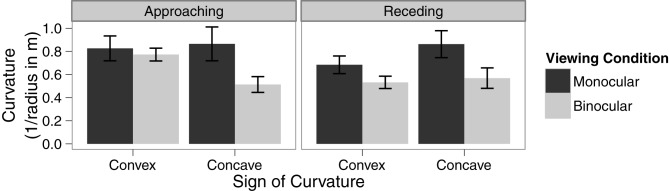
**Means in each condition with error bars representing ±1 between-subjects standard error of the mean**.

A within-subjects ANOVA was conducted to analyze the effect of viewing condition, type of curvature, and direction of motion on the threshold of discrimination between linear and curved trajectories. There was a significant main effect of viewing condition, *F*_(1, 15)_ = 13.73, *p* = 0.002, η^2^_G_ = 0.08. The main effects of type of curvature and direction of motion were not significant, *p* = 0.99 and 0.22, respectively. However, there was a significant interaction between viewing condition and type of curved trajectory, *F*_(1, 15)_ = 6.64, *p* = 0.02, η^2^_G_ = 0.02. There were no other interactions (see Figure 3 for details). Paired sample *t*-tests revealed that the effect of viewing condition is statistically significant for concave curves, *t*_(15)_ = 4.37, *p* < 0.001, but not for convex curves, *t*_(15)_ = 1.49, *p* = 0.16. Notably, the main effect of direction of motion, was not significant and there were no significant interactions involving direction of motion, all *p*s > 0.09. This suggests that the temporal order of the availability of disparity information does not have a strong effect on the detection of a curved trajectory.

## Discussion

The purpose of the present experiments was to examine the ability of observers to detect curved motion paths from monocular information sources and to assess whether binocular information improved detection performance. In Experiment 1 we examined the effect of viewing condition (binocular vs. monocular) and type of curvature (concave vs. convex) on the perception of motion trajectories. We found that detection thresholds were lower when disparity information was present and the motion path was a concave trajectory. In contrast, the presence of disparity information did not result in lower thresholds for convex trajectories.

One source of binocular information that differs for approaching convex and concave motion trajectories is the maximum rate of change in disparity, which is determined both by the distance of the object and by the rate of change in depth of the object. For concave trajectories, the maximum rate of change in depth occurred at the end of the motion trajectory when the object was at the closest distance to the observer. For convex trajectories, the maximum rate of change in depth occurred at the beginning of the motion trajectory when the object was at the greatest distance from the observer. Thus, the maximum rate of change in disparity for concave trajectories was larger than for convex trajectories in Experiment 1. The failure to find an effect of disparity for convex motion trajectories in that experiment may be due to the smaller maximum rate of change in disparity compared to concave trajectories.

A second possibility is that the usefulness of the rate of disparity change might be affected by when it occurs relative to the onset of motion. For convex trajectories the maximum rate of disparity change occurred close to the beginning of the motion, whereas for concave trajectories the maximum rate of disparity change occurred toward the end of the motion path. To examine these possibilities we conducted a second experiment in which the motion paths were repositioned to a closer distance and we examined the direction of motion (approaching vs. receding). We found that the direction of motion did not significantly affect detecting convex or concave trajectories. Thus, we failed to find evidence that the results of Experiment 1 were due to a relation between the onset of motion and when the maximum rate of disparity change occurred.

We also found that at the reduced simulated viewing distances in Experiment 2, the availability of disparity information resulted in greater detection performance for both convex and concave motion paths under both binocular conditions. This finding suggests that the failure to find an effect of viewing condition for convex trajectories in Experiment 1 was likely due to the lower rate of disparity change for the convex motion paths.

In both Experiment 1 and 2 observers were able to detect both concave and convex motion paths from monocular information sources. This suggests that changes in the angular speed and angular size of an object's projected image may be used to detect curved trajectories without CDOT and IOVD information.

The results of these experiments demonstrate the ability of human observers to use disparity information and other cues to detect curved motion trajectories. An important question is what neurophysiological mechanisms are involved in processing motion in depth when binocular information is available. Initial research found evidence that neurons in MT or MT+ responded to both motion and disparity (Ponce et al., 2008; Rokers et al., 2009) whereas other studies found evidence that the dorsal ventral area (V3B/KO) were involved in processing motion and disparity (Ban et al., 2012). However, fMRI research found that an occipito-temporal region, anterior to hMT+, responded to stereomotion in depth (Likova and Tyler, 2007). These results, considered together, suggest activation of multiple cortical regions for processing binocular motion in depth that are likely to be important for discriminating 3D motion trajectories. Other research has shown that local velocity in combination with binocular disparity is needed to determine the changing 3D position of a moving object, suggesting that this information is integrated with motion and disparity inputs late in the visual processing pathway (Lages and Heron, 2010).

Considered in a more general context, our results show that the effectiveness of information can vary by distance, with effectiveness of disparity information decreasing with increased distance. Therefore, the utility of information for detecting curved motion paths will vary by distance. This finding has important implications for understanding of visual information required for different action tasks. For example, action tasks such as catching are more likely to use visual information such as disparity because the motion of the object is at relatively close distances (less than 25 m). In contrast action tasks such as detecting the curved trajectory of an approaching object during driving, where the critical information to perform a response will be a greater distances (e.g., greater than 25 m), are less likely to use information such as disparity. Indeed, Cutting and Vishton (1995) provide an excellent overview of the utility of different information sources for depth as a function of distance, and conclude that disparity is only effective for distances less than 25 m. However, consider a driving action task (detect an impending collision) and the driver is travelling at 25 mph and a vehicle is approaching at 25 mph^1^. Under these conditions the driver will have approximately 1 s to respond when the distance of the approaching vehicle is 25 m. As a result, action tasks such as detecting the curved trajectory of an approaching object during driving are more likely to rely on monocular motion information than disparity information. These observations suggests that the utility of information for detecting motion paths will vary according to the distances in which sufficient time is available to initiate and complete an action.

### Conflict of interest statement

The authors declare that the research was conducted in the absence of any commercial or financial relationships that could be construed as a potential conflict of interest.

## Footnotes

^1^It is important to note that detecting the 3D trajectory of an object from motion and disparity is different from detecting a curvilinear trajectory during self-motion (Li and Cheng, 2011; Cheng and Li, 2012)
